# Concentration of Potentially Toxic Elements in Farmed Fallow Deer Antlers Depending on Diet and Age

**DOI:** 10.3390/ani13223468

**Published:** 2023-11-10

**Authors:** Katarzyna Tajchman, Aleksandra Ukalska-Jaruga, Fracisco Ceacero, Pawel Janiszewski, Monika Pecio

**Affiliations:** 1 Department of Animal Ethology and Wildlife Management, Faculty of Animal Sciences and Bioeconomy, University of Life Sciences in Lublin, Akademicka 13, 20-950 Lublin, Poland; 2 Department of Soil Science Erosion and Land Protection, Institute of Soil Science and Plant Cultivation—State Research Institute, Czartoryskich 8, 24-100 Puławy, Poland; 3 Department of Animal Science and Food Processing, Czech University of Life Sciences Prague, Kamycka 129, 165 00 Prague, Czech Republic; 4 Department of Fur-Wearing Animal Breeding and Game Management, University of Warmia and Mazury in Olsztyn, Oczapowskiego 2, 10-719 Olsztyn, Poland

**Keywords:** *Dama dama*, ecotoxicology, ICP-MS, winter food, pasture plants, antlers

## Abstract

**Simple Summary:**

The rapidly growing antlers of farmed fallow deer (*Dama dama*) reflect the composition of their diet, and furthermore, the risk of toxic substances. The concentration of eight potentially toxic elements, PTE (Cd, Pb, As, Ba, Ni, Sr, La, Ce), was examined in the proximal, middle, and distal positions of the antlers and in the winter food and pasture food that the animals consumed during the increase in individual antler fragments depending on the age. The research was conducted on males aged 2 to 8 years. The oldest fallow deer had the highest amounts of As, Ba, and Sr in antlers. Increasing the body weight of animals and the weight of antlers resulted in a decrease in the concentration of Ba and Sr in tissues. The highest amounts of Cd were in food in June, and Ba in spring and winter. The obtained research results can be used by farmers to make seasonal decisions regarding nutrition. Breeders can produce food in areas with less pollution and harvest to prepare winter feed at a time when emissions and pollutions are lower.

**Abstract:**

Deer antlers, usually harvested annually on a farm, are an accessible material used to determine the exposition to potentially toxic elements, PTEs, during growth. Moreover, the study of antlers from animals of different ages allows the assessment of long-term exposition to these elements. The aim of the study was to analyze the concentration of eight potentially toxic elements (Cd, Pb, As, Ba, Ni, Sr, La, Ce) in individual positions of the antlers (first, second, and third position, corresponding to the stages of development and life of these animals) and in the food that the animals consumed during the growth of individual antler fragments, depending on the age of the farmed fallow deer (*Dama dama*). The mineral composition of samples was analyzed using inductively coupled plasma mass spectrometry (ICP-MS). The analysis included 31 male deer aged 2–8 years old. The average concentration of Pb, Ba, and Ni was higher in the second position of the antler, and As, La, and Ce in the third position. In addition, the oldest individuals showed a higher Cd, Pb, and As concentration in the third position. A significant positive relationship was found between the age of animals and accumulation of As (r = 0.582, *p* < 0.05), as well as Ba and Sr (r = −0.534, r = −0.644 at *p* < 0.05, respectively). The average content of Ba and Sr also significantly negatively depended on body mass and antler mass stags (r = −0.436, r = −0.515 at *p* < 0.05, respectively). Cd concentration in feed was significantly higher in June compared to winter, spring, and later summer (*p* < 0.05). On the other hand, the concentration of Ba in food was significantly higher in spring and winter than in early and later summer (*p* < 0.05). An increase in the PTEs in the pasture determined the concentration of these components in fallow deer antlers.

## 1. Introduction

Wild animals are often used as bioindicators of environmental pollution due to their natural exposure to negative habitat factors [1,2,3,4]. Cervid antlers, usually collected annually as hunting trophies or clipped during a typical farm zootechnical action, can be readily available material used to determine the direct exposure of animals to pollutants. Antlers, as bone tissue that develops over a certain period (usually 120 days), accumulate potentially toxic elements (PTEs) by incorporating and retaining them in bone structures [5,6,7]. Therefore, this material, especially the basal sections, is a valuable source of information on direct exposure and bioavailability of these compounds through the diet of animals living in a given area, indirectly indicating environmental contamination [8,9,10,11,12]. However, the mineral requirements increase during the antler growth, and the deer diet is not enough to meet this high demand, leading to skeletal resorption [13] and the so-called physiological exhaustion [14,15]. Minerals such as calcium, phosphorus, and other elements that have previously been deposited in the bones are therefore transported through the circulatory system from the bones to the antlers [16]. This way, the last sections of the antlers may also accumulate PTEs with which the animal had been in contact throughout its life. Similarly, the comparison of antlers from animals of different ages allows for the assessment of long-term bioaccumulation of certain PTEs and the level of exposition to environmental pollution. Interference between calcium and the enhanced uptake of PTEs has been shown, especially Pb, whereby the low Ca status of food and animals promotes the uptake of potentially dangerous substances in animal tissue [17]. Due to the seasonally limited lifespan of antlers, the concentrations of these pollutants in different sections indicate the accumulation of pollutants in a specific time interval (from a few weeks to a dozen) particular to the species [8,9,10,11,12]. In addition, antler mineral composition is not obscured by remodeling effects [16].

PTEs penetration occurs mainly through the digestive tract [18,19,20,21,22]. Farmed cervids are likely to be less exposed to pollution compared to wild animals because they live in a limited area and cannot translocate and feed, e.g., roadside vegetation [23,24,25]. However, fodder of farm origin and pastures may include different compounds from fertilizers and secondary pollutants like dust from atmospheric deposition. Studies conducted on European roe deer living in areas characterized by increased concentrations of Pb and/or F^−^ showed that drastically increased exposure to harmful minerals affected the quality of the animal antler [12]. The average weight of these organs drastically decreased even up to 2–3 years after exposition to the toxic agent. Therefore, the weakening of the antler mineralization and the growth rate may result in a higher frequency of antler breakage than those from “uncontaminated” areas [12]. Previous studies on farmed deer from northeastern Poland showed that the presence of PTEs in bone and marrow tissues have been observed in the animals, despite the absence of environmental pollution [22]. However, there is evidence that even uncontaminated areas may cause the impairment of mineralization in antlers under the influence of cervids eating insufficiently nutrient-rich food [9]. Therefore, the antlers can be seen as samples containing information on the exposure of deer to pollutants present in their habitat.

This is the first detailed study focusing on the link between habitat pollution (food consumed by farmed fallow deer on pastures) and the accumulation of PTEs in individual sections of antlers corresponding to the stages of development and life of these animals. The PTEs content in the antlers can be valuable information for farmers who can take action to provide food with a lower content of contaminants in the winter and to ensure clean pastures in the summer. In addition, the content of the tested substances in the antlers shed every year by animals informs potential consumers that the body of deer may also contain these substances, most likely by accumulation throughout life. The following research hypotheses were adopted: (1) PTEs that bioaccumulate in the internal bones throughout life will appear in higher concentrations in older animals, (2) their concentration will also be higher in the top of the antler due to the mobilization of skeletal resources, and (3) the concentration of PTEs in the environment and, thus, in food affects their content in the basal and middle parts of the antlers. Thus, this study is based on the analysis of the of eight PTEs concentrations considered the most toxic to the environment (cadmium—Cd, lead—Pb, arsenic—As, barium—Ba, nickel—Ni, strontium—Sr, lanthanum—La, and cerium—Ce) in three selected positions of the farmed fallow deer (*Dama dama*) antlers as well as in the food that the animals consumed during the period of growth and formation of the antlers.

## 2. Materials and Methods

### 2.1. Experimental Design

The study was carried out on the same animals as in Tajchman et al. [15]. The farmed fallow deer were extensively bred at the Research Station of the Institute of Parasitology, Polish Academy of Sciences, Kosewo Górne, in Poland. The research was based on tissue analysis of 31 stags in the age range from 2 to 8 years old, divided into four groups: (I) 8 individuals, fourteen months old, average body weight = 54.40 kg, average antler mass = 0.02 kg; (II) 6 three-year-olds, average body weight = 63.33 kg, average antler mass = 0.29 kg; (III) 7 four/five-year-olds, average body weight = 83.71 kg, average antler mass = 0.70 kg; group (IV) 10 six/eight-year-olds, average body weight = 93.90 kg, average antler mass = 0.76 kg. The breeding system was based on the methodology of DEFRA [26], FEDFA [27], and Mattiello [23]. The nutrition of each animal was the same throughout its life. Generally the diet was varied but based mainly on the high protein content necessary for the proper development of deer. On average, each animal ingested Josera Phosphoreimer multi-ingredient licks (Josera, Nowy Tomyśl, Poland) as well as about 260 g per day of a mixture comprising 70% oats, 15% rapeseed concentrate (33% crude protein; Eko-pasz, Mońki, Poland), and 15% of soybean concentrate (with 45% crude protein content; Eko-pasz, Mońki, Poland).

### 2.2. Sampling

Antler samples were taken from the positions similar to in the research of Tajchman et al. [15] Briefly, antlers were cut about 1 cm above the burr, for safety reasons, once antlers were dead bone, and stripped of velvet skin. The process was performed with a handsaw in order to make precise cutting. During the procedure, all fallow deer were subject to routine management and maintained in good health and body condition with standard procedures that minimized the sense of stress. These are routine activities performed yearly in farmed stags, and lead to the prevention of fights between males and, thus, the restriction of possible falls and injuries among the handlers.

Three samples of the winter feed (10 g each), entirely secured for the winter, were taken from the above-described mixture and hay. Plant samples from pastures were collected from 2 summer pens with an area of 4.5 ha and 9.15 ha, located in a homogeneous habitat where males of farmed fallow deer stayed from April to November. In early spring, representative areas of three in each pen with an area of 1 m^2^ were fenced off so that the animals could not take up biomass, but in places where they foraged most often. Plant samples were collected on three dates from the pens: at the beginning of the pasture period (April/May—first term), in early summer (June—second term), and in late summer before ossification of the antlers (July—third term). In winter, they were not collected for plant analysis because they were almost nonexistent or were covered with snow. Three samples were collected from each pen and on each term, as described by Kulik et al. [28].

### 2.3. Analysis of PTEs Concentration in Antlers, Winter Food, and Pasture Plants

The analysis of PTEs concentration was conducted using inductively coupled plasma mass spectrometry (Agilent quadrupole 7500CE ICP-MS) following protocols widely used in recent years for analyzing antler samples [15] and winter food and plants pasture [28]. The extracts were prepared in concentrated nitric acid by microwave digestion. A blank sample and certified reference material (NIST1400 and CRM028-050) were included in the analyses for quality control of the entire analytical process. The basic validation of the parameters included the recognition of recovery, ranging from 90 to 97%, and precision, defined as a relative standard deviation < 3%. The limit of detection (LOD) ranged from 0.007 mg/kg to 0.099 mg/kg.

The study did not determine the absorption of PTEs since it would be challenging to determine the excretion of the tested substances in an extensive rearing system of cervids based on rotational grazing.

### 2.4. Statistical Analysis

The analyzed variables are shown using the mean and standard deviation. The normality of the distribution of variables in the research groups was verified using the Shapiro–Wilk normality test. The Kruskal–Wallis test was used to test the differences between the groups, and pairwise comparisons were made with the Mann–Whitney test with Bonferroni correction. The Spearman’s rank correlation test estimated the relationship between body weight, antler weight, age, and minerals. The relationship between PTEs in winter fodder and pasture vegetation from different terms, as well as between individual antler positions, was calculated using ANOVA analysis of variance. A significance level of *p* < 0.05 was established to indicate the existence of statistically significant differences or relationships. The statistical analyses were carried out using the Statistica 9.1 computer software (StatSoft, Poland). Principal component analysis (PCA) was additionally used to determine the relationship between the variables expressed by the average PTEs content in the antlers and the type of supplemented food. Additionally, cluster analysis using the k-means method allowed the separation of homogeneous groups within the analyzed variables.

## 3. Results

The average concentration of some elements was clearly higher in the second position of the antler (Pb, Ba, Ni), while for others it was higher in the third position (As, La, Ce). In addition, the oldest stags (group IV) showed a higher concentration in position 3 of the antler compared to the others (Cd, Pb, and As; Table 1).

There was a significant relationship between the tested groups of animals in the case of As, Ba, and Sr (*p* < 0.05). A significantly higher average As concentration was found in the oldest fallow deer (group IV) compared to the younger ones (group II) (*p* = 0.007). On the other hand, a significantly higher average Ba content was obtained in animals from group II compared to group IV (*p* = 0.022). A significantly high mean Sr concentration was found in younger fallow deer (group II) compared to older individuals (group III and IV, *p* = 0.047 and *p* = 0.003, respectively) (Table 2).

Moreover, the measurements of PTEs from individual positions (first, second, third positions) between groups of the studied animals were also analyzed (Table 3). A significant relationship was found between groups I–III, I–IV, II–III, and II–IV in position 1 of the antlers and between groups II–IV in the second and third positions (*p* < 0.05). For the concentration of Ba, a significant relationship was observed between groups II–IV in the second and third positions (*p* < 0.05). In addition, a significant positive relationship was found between groups I–II and I–III only in the first position of the antler for Ni concentration (*p* < 0.05). A significant relationship was found of the average Sr content between groups I–IV and II–IV in the first position of the antlers and between groups II–IV in the second position and between groups II–III and II–IV in the third position of the antlers (*p* < 0.05). In addition, a significant positive relationship was found between groups II–III in the second and third positions of the antlers and between groups II–IV in the second position for La (*p* < 0.05).

Correlations between PTEs concentration and age, body, and antler mass were also analyzed (Table 4). A significant positive relationship was found between As and the age of animals, and a negative relationship between Ba and Sr and age (*p* < 0.05). The average content of Ba and Sr negatively correlated with body and antler mass (*p* < 0.05).

The concentration of PTE among antler positions was compared (Table 5). There was a significant difference in Ba, La, and Ce content (*p* < 0.05). There were significant differences in Ba concentration between the first and third positions and between the second and third positions (*p* = 0.013 and *p* = 0.019, respectively). The content of Ba was the lowest in the third position of the antler compared to the first and second positions. Moreover, a statistically significant difference in the concentration of La and Ce was found between the second and third positions of the antler (*p* = 0.047 and *p* = 0.006, respectively). The highest concentration of La and Ce was in the third position of the antler (Table 5).

The concentration of PTEs in winter food and pasture vegetation was analyzed in three terms corresponding to the growth of individual fragments of the antler positions of farmed fallow deer bulls. There were statistically significant differences between the examined terms in the concentration of PTEs in the food consumed by the tested animals in the case of Cd, Ba, and Sr (*p* < 0.05). The average Cd concentration was significantly higher in June (second term) compared to the first and third terms. On the other hand, the average concentration of Ba in food was significantly higher in the second and third terms. The average concentration of Sr was significantly higher in the first term. In the third term, there was a similar Sr content to that in the first and third terms (Table 6).

**Figure 1:**
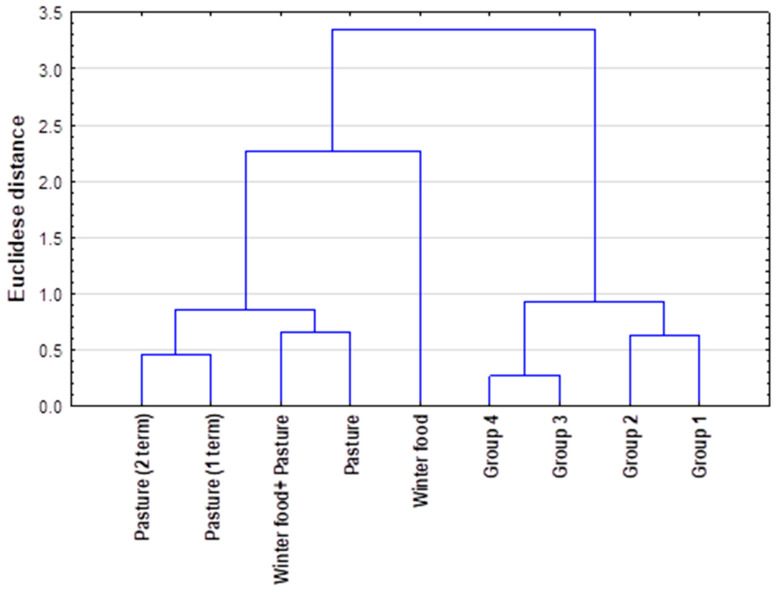
K-means clustering method for analyzed mean concentration of potentially toxic elements in food and antlers.

The k-measures grouping method indicated that the PTEs content in antlers and food differed significantly depending on the samples tested (food vs. antlers), which confirms the forms of occurrence and proportions between PTEs, depending on the matrix (Figure 1). Minerals contained in winter food and pasture are properly assimilated by animals, and only a proportionately small part of them is deposited in the antler tissue.

**Figure 2:**
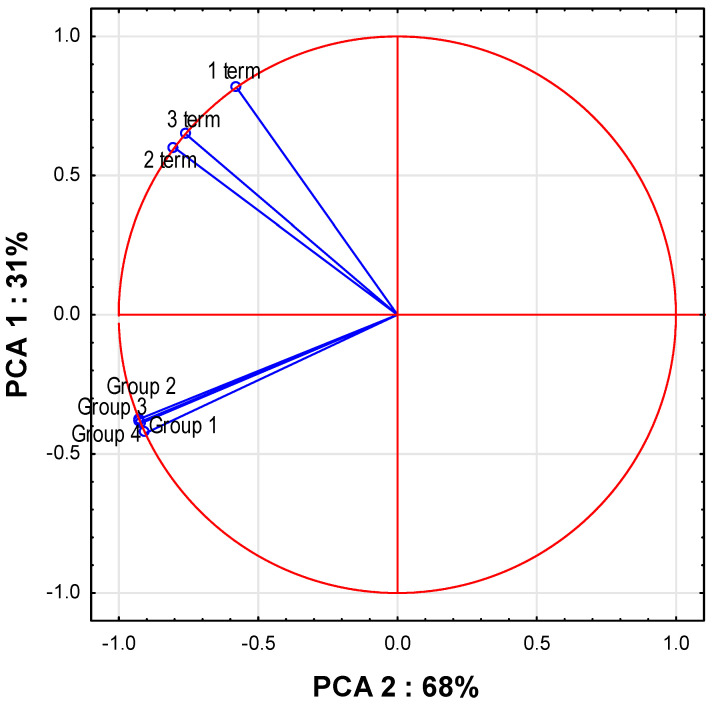
PCA analysis expressed by coordination biplot. Correlation of average metal PTE in antlers and supplemented food variable.

The PCA analysis showed interrelationships between the studied variables. Generally, the PTEs content of foods and antlers depended on two factors that account for 99% of the variability. The PCA 1 factor, characterized by a high correlation value (68%) with the examined variables (r = −0.73 to −0.94), has the greatest impact. Similarly, the PCA 2 factor (31%) is highly positively correlated with PTEs content in antlers (r = 0.64 to 0.67) and negatively correlated with PTEs content in plants, significantly, only for the first term (r = −0.60 and −0.64, appropriately). The results indicate that there may be a greater absorption of PTEs from winter fodder and plants in winter and spring than in summer (Figure 2).

## 4. Discussion

The analyzed PTEs are particularly dangerous for bone tissues due to the interaction of the primary building material (i.e., calcium) with Pb, Cd, As, and Ba [29,30,31,32]. Cd in cells disturbs the metabolism of Ca, Mg, Fe, Zn, and Cu, which leads to demineralization, osteomalacia, and osteoporosis of the bones, and disorders of the body’s regulatory functions in which the participation of these ions is necessary [30,31]. High concentrations of Ca and Fe may provide some protection against lead poisoning, while low concentrations increase susceptibility [33]. In the case of Sr, a previous study shows that it produces an ionic substitution with Ca in bone tissue [34]. It is worth noting that a significantly high mean Sr concentration was found in younger fallow deer compared to older individuals. In younger animals, skeletal growth will be more important than the yearly removed antlers. Thus, keeping as much Ca as possible for the skeleton seems reasonable. Sr can be used in the antler, especially since it can substitute Ca in the hydroxyapatite crystals [35,36]. Changes of this type and, in general, in the relations between the various elements of bone, can lead to changes in the mechanical performance of bone material, some of which may lead to a loss of bone strength [16,37]. Ba is also a calcium agonist and is incorporated into the bone structure instead of Ca. Ba enters bone at a rate up to five times faster than that of Ca or Sr [32]. It has been shown that the highest Ba concentration is found in the bones (about 91% of the total body amount). The penetration of Ba into bone is 1.5 times to 5 times greater than that of Ca or Sr [32]. This process happens very quickly. As early as 24 h after the end of exposure of 56 rats to BaCl_2_ in the form of an aerosol, about 78% of the total Ba in the body was found in the bones and reached the value of 95% on the 11th day after exposure [38]. The rest of the Ba was distributed to soft tissues (brain, heart, kidneys, spleen, pancreas, trachea, and lungs). Animal studies also indicate the potentially toxic effect of lanthanides on fetal development (reduced body weight and delayed sexual maturation and eye opening) [39].

A significant source of Cd in the environment is artificial fertilizers (e.g., superphosphates), which are contaminated with this metal in amounts of 10 to 100 mg kg^−1^. The largest quantities of As are supplied to the body with drinking water, while its content in food products depends on the origin of the raw material and its type. In countries where drinking water comes from groundwater, the estimated amount of As supplied is in the range of 1–5300 µg L^−1^. This value is over a hundred times higher than the limit value set by FAO/WHO experts [40]. Long-term and widespread use of fertilizers leads to soil contamination with heavy metals, including Cd [41,42].

It is worth emphasizing that As and Pb were the ingredients used until the late 1960s for debarking agents on forest wildlife in some herbicides and silvicides [43]. Due to its salty taste, small amounts are highly toxic to deer. Webb et al. [44] showed that treated wood threatened wildlife if ingested 4 to 7 days post-treatment. Dickinson [45] reported that the oral lethal dose (100% mortality) for cattle is 80 to 100 mg MSMA kg^−1^ body weight. The liver and kidney tissue of one animal succumbing on day 10 contained 24 and 64 ppm arsenic residue, respectively. The antlers of the study fallow deer contained much lower amounts of As; however, due to the short time of their growth, there were high concentrations of this element in the distal parts of the bone tissue. In turn, the concentration of Cd in the antlers of fallow deer was similar to that observed in the bones of red deer inhabiting Northwestern and Central Europe (0.004–0.018 mg kg^−1^), while Pb was much lower (2.20–3.70 mg kg^−1^). Similarly, the content of Cd (0.13 mg kg^−1^) was similar, while Pb (0.97 mg kg^−1^) was much lower in the deer diet [17].

Ba is used in the pharmaceutical industry and medicine. It is used as a contrast agent because X-rays do not penetrate it. It is used as a pigment in the production of paints, a weighting agent in the production of paper (photo paper), a filler for rubber and plastics, and in cosmetics to produce powders. Ba is in the group of elements with an undetermined biological role. Once considered toxic and harmful to health, some elements turned out to be very necessary for some immunological processes [46]. La and Ce are of great importance in producing alloys, fuel cells, and nickel metal hydride batteries. In addition, they are essential in electronics; they are used as tracers in medicinal applications, fertilizers, and water treatment. Small amounts of La or Ce from the environment or properly administered seem to have no harmful effects, while higher doses have produced harmful effects, especially in the organs they accumulate (lungs, liver) [39,47].

The content of most PTEs (Pb, As, Ba, Ni, Sr) was higher in the proximal parts of the antlers of fallow deer (i.e., more linked to the diet), and Cd, As, La, and Ce were higher in the distal parts (i.e., more linked to long-term exposition and bioaccumulation in internal bones and subsequent mobilization for supporting antler growth). Some, such as Pb, Ba, and Sr, were accumulated to a greater extent in the antlers of younger animals, but Cd, La, or Ce were found to have accumulated more in older ones. The fact that the same PTEs prevailing in the distal parts are also found in higher concentrations in older animals further supports our hypotheses. Although the highest concentrations of all analyzed PTEs were found in pasture vegetation compared to the concentrated mixture based on cereal grains, most of them (Cd, Pb, Ba, Sr, La) had the highest concentration in the second term (June); only As, Ni, and Ce concentrations were higher in the first term (April). It is possible that this was due to greater pollution resulting from increased vehicular traffic during the holiday season and spring fertilization of pastures. However, most likely, the lack of a trend in the accumulation of PTEs in antlers related to the content of these substances in food could be caused by their displacement from the skeletal system, especially since previous studies on farmed deer showed a high concentration of these substances in bones and the skeletal marrow (22). Only the accumulation of Cd and As in older fallow deer was higher in antlers than in deer bones, while Pb, Ba (except group II in second positions and group III in first), and Ni were higher in bones. It should also be emphasized that the bones came from only 6/7-month-old animals but already contained large amounts of Pb, Ba, and Ni (0.545 mg kg^−1^, 87.978 mg kg^−1^, and 1.667 mg kg^−1^, respectively) [22].

However, it should also be emphasized that antlers grow rapidly from their pedicle (base) while in velvet during the spring and summer, as fast as 3/4 inch/week for yearlings and 1/2 inches per week for adults during peak growth [48]. The growth rate slows dramatically during late summer while the mineralization of the antler is completed. We analyzed the first and second positions in antlers, which represent the period of fastest growth and mineral requirements. The antler is still growing in length, and at the same time, it is already mineralizing in the base/middle, so it is very demanding with maximum blood flow, hence the high concentrations of some PTEs in its proximal positions.

Studies conducted on wild deer living in the same area as farmed fallow deer showed higher concentrations of Cd in the liver, kidneys, and muscles but similar or higher Pb in the antlers [2]. On the other hand, a higher concentration of Cd and Pb (except for group II in the second position and group III in the first one) was found in the hair of roe deer from the nearby Kuyavian–Pomeranian province. However, the hair cover is also periodically replaced [49]. The reasons should be sought from the variability of the accumulation of individual trace elements in a given tissue and the fact that wild animals take food from various sources. In the antlers of wild roe deer collected in Wrocław Districts, Międzylesie Woodlands, Niemcza District, and Białowieża Forest, higher concentrations of Cd and Pb were found than those of farmed fallow deer [8]. It can therefore be concluded that farmed deer are less exposed to environmental contamination compared to wild ones.

The data generated by this study could be used by farmers to make seasonal decisions about feeding. Breeders can try to produce food in areas with lower pollution or harvest crops at a time when emissions are lower, at least in the case of preparation of winter feed.

## 5. Conclusions

In summary, the concentration of some PTEs, especially As, in the antlers of farmed fallow deer increased significantly with their age, while Ba and Sr decreased. The highest concentration of As, La, and Ce was shown in the distal (third) position, but Pb, Ba, and Ni were shown to be highest in the antler’s middle (second) position. Increased PTEs in pasture food determined the concentration of these components in fallow deer antlers, especially in the basal sections. However, for elements like Cd, As, La, and Ce, it seems to be mainly caused by the long-term accumulation in the bones and further transportation during the most demanding periods of antler growth when minerals are mobilized from the skeleton to the antler. The obtained results indicate that in winter and spring, there may be a greater absorption of PTEs from winter fodder and plants than in summer, which is related to the growth rate of the examined organs. This may be due to higher emissivity due to combustion processes during heating periods, and, associated with this, a greater release of harmful compounds into the atmosphere, which, together with dust, fall on the soil, as well as a result of spring fertilization of pastures.

## Figures and Tables

**Table 1:** Mean concentration (M) and standard deviation (SD) of PTE in the 1st, 2nd and 3rd positions of antlers of farmed fallow deer.

Analysed Parameters		Group I Position 1		Group II						Group III						Group IV					
				Position 1		Position 2		Position 3		Position 1		Position 2		Position 3		Position 1		Position 2		Position 3	
		M	SD	M	SD	M	SD	M	SD	M	SD	M	SD	M	SD	M	SD	M	SD	M	SD
Cd	mg/kg	0.004	0.003	0.008	0.003	0.015	0.014	0.015	0.014	0.014	0.009	0.013	0.09	0.014	0.012	0.011	0.011	0.007	0.006	0.029	0.045
Pb		0.166	0.050	0.197	0.083	1.375	2.008	0.385	0.359	0.698	1.112	0.209	0.081	0.286	0.142	0.317	0.276	0.326	0.313	0.414	0.322
As		0.024	0.013	0.023	0.017	0.032	0.042	0.061	0.060	0.085	0.032	0.070	0.026	0.076	0.027	0.136	0.135	0.090	0.020	0.113	0.039
Ba		68.704	13.115	77.533	7.173	81.950	8.628	71.532	3.758	70.943	27.282	61.711	15.422	53.881	18.383	59.211	11.174	54.782	10.530	53.411	13.903
Ni		0.253	0.092	0.431	0.137	0.853	1.004	0.594	0.396	0.511	0.190	0.382	0.124	0.420	0.262	0.400	0.118	0.411	0.109	0.523	0.196
Sr		5.571	0.734	5.641	0.441	5.469	0.343	5.223	0.238	4.844	0.994	4.556	0.660	4.329	0.711	4.394	0.237	4.269	0.349	4.268	0.421
La		0.021	0.014	0.015	0.005	0.019	0.009	0.028	0.011	0.014	0.005	0.010	0.002	0.017	0.007	0.017	0.013	0.012	0.004	0.032	0.048
Ce		0.054	0.033	0.030	0.012	0.040	0.017	0.064	0.025	0.034	0.011	0.022	0.004	0.041	0.016	0.036	0.028	0.026	0.011	0.062	0.081

**Table 2:** Comparison of the mean measurements from three antler positions between the three age-class groups of animals.

Analysed Parameters		Group II		Group III		Group IV		H_(3,23)_	*p*	ρ
		M	SD	M	SD	M	SD			
Cd	mg/kg	0.013	0.005	0.013	0.005	0.015	0.015	0.337	0.845	-
Pb		0.653	0.643	0.398	0.355	0.352	0.202	0.284	0.867	-
As		0.039	0.034	0.077	0.021	0.113	0.047	9.321	0.009 *	II–IV (0.007 *)
Ba		77.005	4.467	62.178	19.961	55.801	10.411	7.906	0.019 *	II–IV (0.022 *)
Ni		0.626	0.462	0.438	0.091	0.445	0.059	0.112	0.945	-
Sr		5.445	0.279	4.576	0.703	4.311	0.285	10.794	0.004 *	II–III (0.047 *), II–IV (0.003 *)
La		0.021	0.003	0.014	0.003	0.020	0.016	4.465	0.107	-
Ce		0.044	0.007	0.032	0.008	0.041	0.028	3.653	0.161	-

M—mean, SD—standard deviation, H—Kruskal-Wallis test, ρ—Spearman’s rank correlation coefficient, * values statistically significant at *p* < 0.05.

**Table 3:** Between groups comparison of measurements of potentially toxic elements at each studied position.

Analysed Parameters	Position 1			Position 2			Position 3		
	H_(3,31)_	*p*	ρ	H_(3,23)_	*p*	ρ	H_(3,23)_	*p*	ρ
Cd	11.889	0.007 *	I–III (0.003 *)	0.899	0.637	-	0.298	0.861	-
Pb	6.790	0.078	-	0.073	0.964	-	0.494	0.781	-
As	22.505	0.0001 *	I–III (0.014 *), I–IV (0.0009 *), II–III (0.025 *) II–IV (0.002 *)	7.165	0.027 *	II–IV (0.023 *)	6.290	0.043 *	II–IV (0.046 *)
Ba	6.957	0.073	-	9.787	0.007 *	II–IV (0.005 *)	7.455	0.024 *	II–IV (0.035 *)
Ni	13.031	0.004 *	I–II (0.045 *), I–III (0.006 *)	1.115	0.572	-	1.989	0.369	-
Sr	15.082	0.001 *	I–IV (0.012 *), II–IV (0.009 *)	10.628	0.004 *	II–IV (0.003 *)	10.454	0.005 *	II–III (0.027 *), II–IV (0.006 *)
La	1.866	0.601	-	7.494	0.023 *	II–III (0.046 *), II–IV (0.043 *)	6.624	0.035 *	II–III (0.046 *)
Ce	4.974	0.174	-	6.717	0.034	-	5.019	0.081	-

H—Kruskal-Wallis test, ρ—Spearman’s rank correlation coefficient, * values statistically significant at *p* < 0.05.

**Table 4:** Relationship between the concentration of potentially toxic elements on age, body mass and antler mass of farmed fallow deer.

Analysed Elements *N* = 23	Age		Body Mass		Antler Mass	
	ρ	*p*	ρ	*p*	ρ	*p*
Cd	−0.156	0.477	−0.005	0.980	−0.147	0.500
Pb	−0.081	0.713	−0.159	0.466	−0.205	0.346
As	0.582	0.003 *	0.340	0.112	0.389	0.066
Ba	−0.534	0.008 *	−0.436	0.037 *	−0.648	0.0008 *
Ni	−0.041	0.849	−0.006	0.976	−0.124	0.571
Sr	−0.644	0.0008 *	−0.515	0.012 *	−0.582	0.003 *
La	−0.395	0.061	−0.112	0.610	−0.322	0.133
Ce	−0.407	0.053	−0.019	0.930	−0.278	0.198
**Measurements’ correlations**						
Age	-	-	-	-	-	-
Body mass	0.663	<0.001 *	-	-		
Antler mass	0.665	<0.001 *	0.727	<0.001 *	-	-

ρ—Spearman’s rank correlation, * values statistically significant at *p* < 0.05.

**Table 5:** Mean concentration (M) and standard deviation (SD) of potentially toxic elements in fallow deer antlers at each studied position.

Analysed Parameters		Position 1		Position 2		Position 3		F	*p*	ρ
		M	SD	M	SD	M	SD			
Cd	mg/kg	0.011	0.009	0.011	0.010	0.021	0.032	0.177	0.915	-
Ba		66.215	18.376	62.803	16.174	57.049	15.594	13.238	0.001 *	1–3 (0.013) 2–3 (0.019)
Pb		0.414	0.671	0.608	1.140	0.349	0.255	2.952	0.228	-
Ni		0.434	0.152	0.527	0.553	0.497	0.275	0.095	0.953	-
As		0.099	0.101	0.076	0.030	0.092	0.046	1.143	0.564	-
Sr		4.739	0.704	4.568	0.603	4.468	0.616	1.809	0.404	-
La		0.015	0.009	0.013	0.006	0.025	0.033	9.181	0.010 *	2–3 (0.047)
Ce		0.034	0.021	0.028	0.013	0.053	0055	13.469	0.001 *	2–3 (0.006)

M—mean, SD—standard deviation, F—analysis of variance ANOVA, ρ—Spearman’s rank correlation, * values statistically significant at *p* < 0.05.

**Table 6:** Mean concentration (M) and standard deviation (SD) of potentially toxic elements in the foods.

Analysed Parameters		Winter Foods		Pasture		Winter Foods + Pasture April/May (Term 1)		Pasture				F	*p*	ρ
		Concentrate Feed	Hay	April/May				June (Term 2)		July (Term 3)				
				M	SD	M	SD	M	SD	M	SD			
Cd	mg/kg	0.01	0.07	0.151	0.035	0.121	0.060	0.208	0.089	0.121	0.028	5.597	0.009 *	1–2 (0.018 *), 2–3 (0.021 *)
Pb		0.05	0.21	0.341	0.165	0.286	0.181	0.292	0.124	0.265	0.065	0.106	0.899	-
As		0.01	0.05	0.036	0.027	0.037	0.026	0.031	0.017	0.026	0.012	0.759	0.478	-
Ba		10.78	14.25	14.795	5.181	14.286	6.335	31.278	15.748	28.308	6.997	7.241	0.003 *	1–2 (0.004 *), 1–3 (0.019 *)
Ni		0.24	2.19	3.723	1.601	3.069	1.768	2.456	1.901	2.175	2.768	0.423	0.659	-
Sr		0.16	1.36	1.096	0.365	1.055	0.465	1.646	0.521	1.296	0.344	4.091	0.029 *	1–2 (0.022 *)
La		0.01	0.12	0.169	0.151	0.144	0.134	0.124	0.055	0.095	0.037	0.717	0.497	-
Ce		0.02	0.23	0.306	0.303	0.263	0.265	0.199	0.116	0.171	0.096	0.655	0.527	-

M—mean, SD—standard deviation, F—analysis of variance ANOVA, ρ—Spearman’s rank correlation, * values statistically significant at *p* < 0.05.

## Data Availability

The manuscript was prepared without the use of artificial intelligence, all data are available from the authors.
